# Succinate Dehydrogenase Is a Direct Target of Sirtuin 3 Deacetylase Activity

**DOI:** 10.1371/journal.pone.0023295

**Published:** 2011-08-17

**Authors:** Lydia W. S. Finley, Wilhelm Haas, Valérie Desquiret-Dumas, Douglas C. Wallace, Vincent Procaccio, Steven P. Gygi, Marcia C. Haigis

**Affiliations:** 1 Department of Pathology, The Paul F. Glenn Labs for the Biological Mechanisms of Aging, Harvard Medical School, Boston, Massachusetts, United States of America; 2 Department of Cell Biology, Harvard Medical School, Boston, Massachusetts, United States of America; 3 Department of Biochemistry and Genetics, Angers University Hospital, School of Medicine, and UMR INSERM, U771-CNRS6214, Angers, France; 4 Center for Mitochondrial and Epigenomic Medicine, The Children's Hospital of Philadelphia, Philadelphia, Pennsylvania, United States of America; Auburn University, United States of America

## Abstract

**Background:**

Sirtuins (SIRT1-7) are a family of NAD-dependent deacetylases and/or ADP-ribosyltransferases that are involved in metabolism, stress responses and longevity. SIRT3 is localized to mitochondria, where it deacetylates and activates a number of enzymes involved in fuel oxidation and energy production.

**Methodology/Principal Findings:**

In this study, we performed a proteomic screen to identify SIRT3 interacting proteins and identified several subunits of complex II and V of the electron transport chain. Two subunits of complex II (also known as succinate dehydrogenase, or SDH), SDHA and SDHB, interacted specifically with SIRT3. Using mass spectrometry, we identified 13 acetylation sites on SDHA, including six novel acetylated residues. SDHA is hyperacetylated in SIRT3 KO mice and SIRT3 directly deacetylates SDHA in a NAD-dependent manner. Finally, we found that SIRT3 regulates SDH activity both in cells and in murine brown adipose tissue.

**Conclusions/Significance:**

Our study identifies SDHA as a binding partner and substrate for SIRT3 deacetylase activity. SIRT3 loss results in decreased SDH enzyme activity, suggesting that SIRT3 may be an important physiological regulator of SDH activity.

## Introduction

Protein lysine acetylation has emerged as a widespread post-translational modification. Proteomic surveys of mammalian cells have identified acetylation on nearly 2200 proteins distributed throughout the cell, undermining the previous perception that acetylation is a largely nuclear event [1]. However, our understanding of the factors that control acetylation and the consequences of this modification remain incomplete.

Sirtuins are a family of class III histone deacetylases and/or protein ADP-ribosyltransferases that are emerging as major regulators of non-histone protein acetylation [2]. Mammals possess seven sirtuins (SIRT1-7) that are, like lysine acetylation, distributed throughout the cell. Sirtuins are increasingly implicated in metabolism, stress responses and longevity [2], suggesting that regulation of lysine acetylation by sirtuins can drive important cellular processes. Intriguingly, sirtuin catalytic activity absolutely requires NAD, leading many to speculate that sirtuin deacetylase activity may be tied to the metabolic state of the cell [2]. Moreover, a strikingly high percentage of enzymes involved in intermediate metabolism and energy production are acetylated [1]. Together, these findings suggest that sirtuins may act as nutrient-sensitive modulators of cellular metabolism, and growing evidence supports this hypothesis [2].

Three sirtuins (SIRT3-5) are localized to the mitochondrion and influence mitochondrial energy production, substrate oxidation and apoptosis [3]. Of the three mitochondrial sirtuins, only SIRT3 null (KO) mice display robust hyperacetylation of mitochondrial proteins, indicating that SIRT3 may be a major mitochondrial deacetylase [4]. In fact, SIRT3 has been shown to directly deacetylate and activate numerous mitochondrial proteins, including acetyl CoA synthetase 2 (AceCS2) [5], [6], glutamate dehydrogenase (GDH) [4], [7], isocitrate dehydrogenase 2 (IDH2) [7], [8], long-chain acyl CoA dehydrogenase (LCAD) [9], superoxide dismutase 2 (SOD2) [10], [11], ornithine transcarbamoylase (OTC) [12] and 3-hydroxy-3-methylglutaryl CoA synthase 2 (HMGCS2) [13], and many additional proteins are reported to be hyperacetylated in SIRT3 KO cells (reviewed in ref. [3]). To expand our understanding of how SIRT3 regulates mitochondrial metabolism, we utilized an unbiased proteomic approach to identify SIRT3-interacting proteins. We found that SIRT3 interacts with several subunits of complex II and V of the electron transport chain (ETC), and we established succinate dehydrogenase (SDH), a member of both the ETC and tricarboxylic acid (TCA) cycle, as a *bona fide* SIRT3 substrate.

## Results

### SIRT3 physically interacts with subunits of complex II and complex V

**Figure 1:**
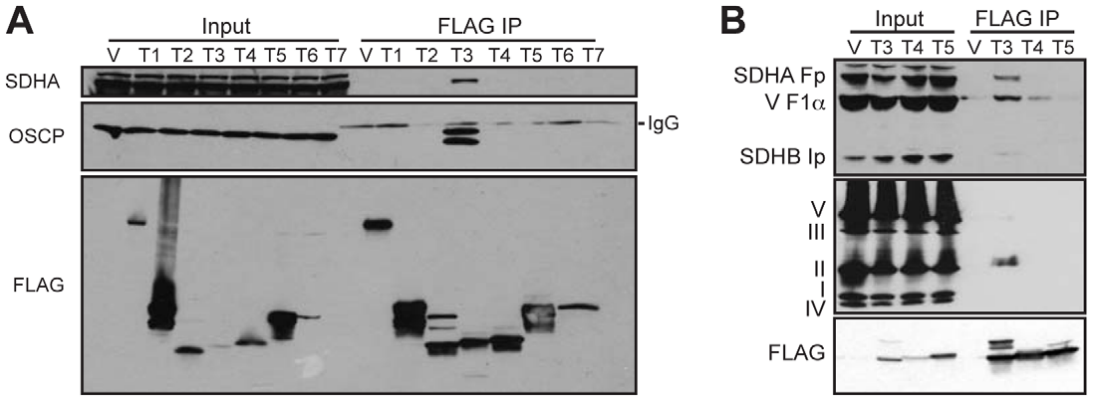
SIRT3 interacts with subunits of complex II and complex V. FLAG-IPs were performed on HEK293T cells transiently expressing FLAG-tagged sirtuins (T1-7). (A) SIRT1-7 IPs were immunoblotted with antibodies against SDHA, OSCP and FLAG. (B) SIRT3-5 IPs were immunoblotted with an antibody cocktail recognizing the SDHA and SDHB subunits of complex II and the F1α subunit of complex V (top) or a cocktail containing antibodies against representative subunits of complexes I–V (middle). SDHB is the subunit recognized by the complex II antibody.

SIRT3 is emerging as major regulator of many acetylated mitochondrial proteins. To uncover potential SIRT3 targets, we performed two independent experiments in which we identified SIRT3-interacting proteins using mass spectrometry (MS) on eluates of FLAG-IPs from HEK293T cells expressing empty vector or SIRT3-FLAG. Candidate interacting proteins were filtered using three criteria: 1) candidate must be identified in both SIRT3 IPs; 2) candidate must not have any peptides identified in control IPs, and 3) candidate must be localized to mitochondria according to the MitoCarta compendium of mitochondrial proteins [14]. Five proteins met these criteria: SIRT3, SDHA, ATP5O/OSCP, HSDL2 and SLC16A1. The two major mitochondrial SIRT3-interacting proteins, SDHA and OSCP, are subunits of oxidative phosphorylation complexes II and V, respectively. We identified additional subunits of complex II (SDHB) and complex V (ATP5A1 and ATP5F1) in SIRT3 IPs, suggesting that SIRT3 may interact with the intact complexes. Furthermore, we identified GDH and subunits of complex I (NDUFA11 and NDUFS8), which were previously identified as SIRT3 targets [4], [7], [15]. Next, we confirmed by immunoblotting that, of the seven sirtuins, only SIRT3 physically interacts with SDHA and OSCP (Fig. 1A). Using two separate antibody cocktail kits, we found that SIRT3 specifically interacts with both the SDHA and SDHB subunits of complex II (Fig. 1B, top and middle panels). We also detected a non-specific interaction between SIRT3 and the F1α subunit of complex V (ATP5A1) (Fig. 1B).

### Many lysines on the surface of SDHA are acetylated

**Figure 2:**
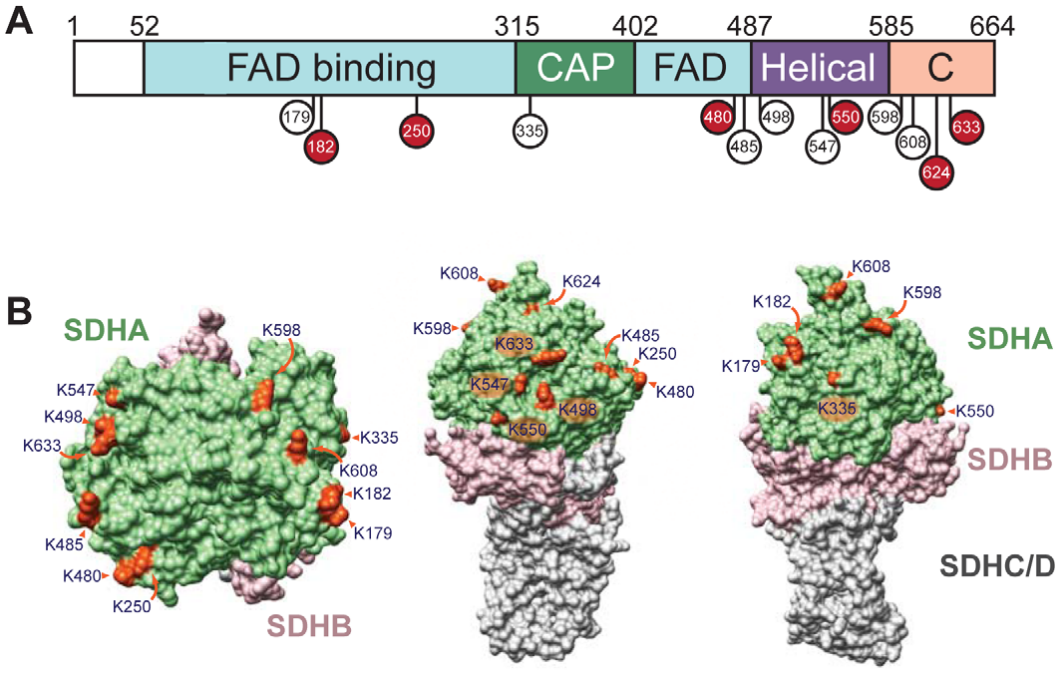
SDHA is acetylated at 13 lysine residues. (A) Schematic of SDHA summarizing the four domains (FAD-binding, capping, helical and C-terminal) with 13 identified acetylated residues shown in red (novel) or white (previously identified). (B) The 13 acetylated lysines were mapped on to the corresponding residues of the crystal structure of avian complex II.

Our IP data indicated that among the seven sirtuins, SIRT3 specifically interacts with both the SDHA and SDHB subunits of complex II. Several independent studies have found that SDHA, but not SDHB, is acetylated [16]–[20]. In order to determine whether SDHA or SDHB could be a target of SIRT3 deacetylase activity, we immunopurified complex II from mouse liver mitochondria and analyzed acetylation by mass spectrometry. We identified a total of 13 acetylated lysines on SDHA and no acetylation on SDHB. Of the 13 sites, 7 were previously identified in mammalian proteomic surveys [16], [18]–[20] (Table S1). We additionally identified K182, K250, K480, K550, K624 and K633 as acetylated residues. SDHA is a highly conserved protein [21], and many of these residues are conserved across metazoans (Table S1). SDHA contains four domains: a FAD-binding domain, a capping domain that closes the active site to solvent, a helical domain and a C-terminal domain [22], [23]. Acetylated lysines were distributed throughout the amino-acid sequence in all four domains of SDHA, with an increase in acetylation toward the C-terminal end of the protein (Fig. 2A), and were found exclusively on the outer surface of the protein (Fig. 2B). The fact that every acetylation site is solute-accessible coupled with the lack of acetylation at the interface between SDHA and SDHB suggests that SDHA may be modified by acetylation after complex II assembly.

Acetylation of SDHA is known to be influenced by fasting, calorie restriction (CR) and SIRT3 expression [17]–[19]. In order to better understand the genetic and physiological regulation of SDHA acetylation, we immunopurified SDHA from liver mitochondria of SIRT3 WT and KO mice, both fed and fasted, in addition to control and CR mice and identified acetylated lysines by MS. To our surprise, we did not detect a simple binary diet or genotype-dependent regulation of modification (Table S1). Interestingly, we did observe that CR *increased* acetylation levels at seven lysines (Fig. S1), consistent with previous reports [18].

### SIRT3 directly deacetylates SDHA

**Figure 3:**
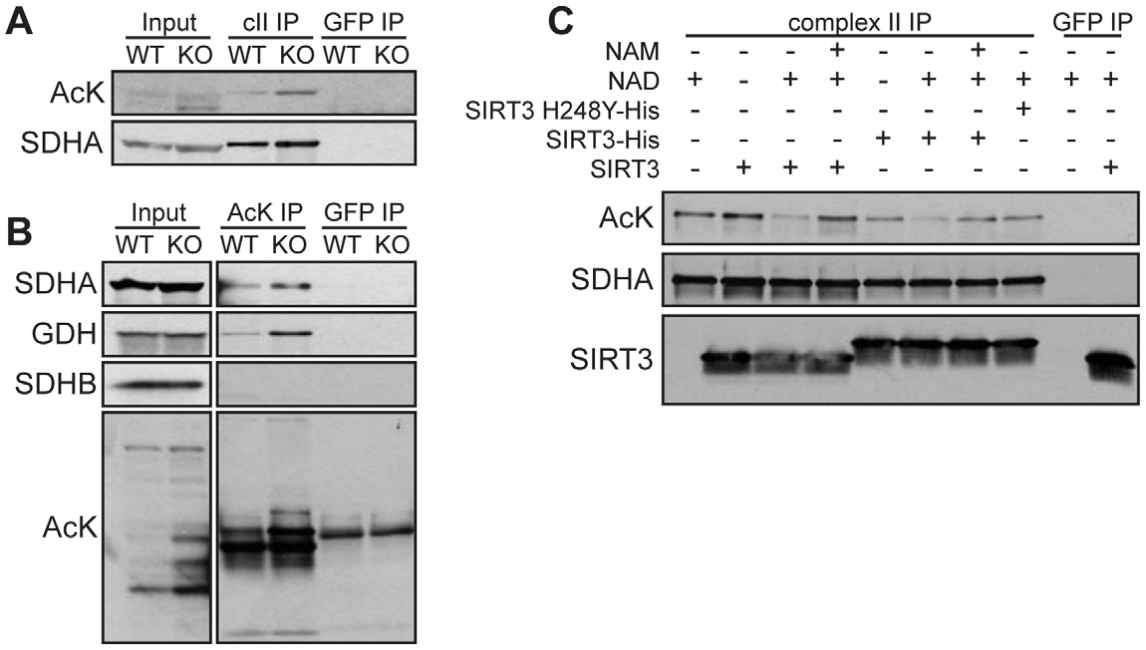
SIRT3 deacetylates SDHA. Complex II (A) and acetylated proteins (B) were immunoprecipitated from liver mitochondria isolated from SIRT3 WT and KO mice. IPs were immunoblotted with antibodies against acetyl-lysine (AcK), SDHA, SDHB and GDH. (C) Complex II immunoprecipitated from mouse liver mitochondria was incubated with recombinant SIRT3 or catalytically inactive SIRT3 (SIRT3 H248Y) in the presence or absence of NAD and NAM, a sirtuin inhibitor. After deacetylation, IPs were immunoblotted using antibodies against acetylated proteins, SDHA and SIRT3. In all panels, antibodies against GFP were used as negative controls.

Having established SDHA as a highly acetylated SIRT3 interacting protein, we next sought to determine whether SIRT3 could regulate the level of SDHA acetylation. A previous study found evidence of increased SDHA acetylation in SIRT3 KO mice [17], but did not analyze acetylation of purified SDHA or the ability of SIRT3 to deacetylate SDHA. To determine whether complex II might be a direct substrate of SIRT3, we first assessed the acetylation status of SDHA in SIRT3 WT and KO mouse liver mitochondria. We found that immunopurified SDHA was hyperacetylated in SIRT3 KO mitochondria (Fig. 3A). (Additional exposures of the complex II IP and inputs from SIRT3 WT and KO liver mitochondria are available in Appendix S1.) Similarly, more SDHA was pulled down by immunoprecipitation of acetylated proteins from SIRT3 KO mitochondria than from WT mitochondria (Fig. 3B). As a positive control, we obtained similar results with GDH, a verified SIRT3 substrate [4], [7]. Using this method, we did not detect acetylation of SDHB (Fig. 3B). Next, we assessed the ability of SIRT3 to directly deacetylate SDHA. We incubated complex II purified from mouse liver mitochondria with recombinant SIRT3 in the presence or absence of NAD and nicotinamide (NAM), a sirtuin inhibitor. SIRT3, but not a catalytically inactive mutant (SIRT3-H248Y), deacetylated SDHA in a NAD-dependent manner (Fig. 3C). Together, these results demonstrate that SDHA is a target of SIRT3 deacetylase activity.

### SIRT3 regulates succinate dehydrogenase activity in vivo

**Figure 4:**
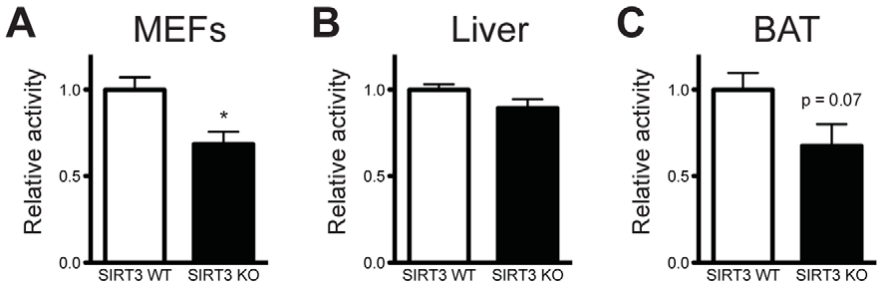
SIRT3 regulates SDH activity. Succinate dehydrogenase activity was measured in (A) SIRT3 WT and KO MEF extracts (*n = 4*), (B) liver mitochondria (*n = 3*) and (C) brown adipose tissue (BAT) mitochondria (*n = 6–7*). SDH activity was normalized to sample protein content and expressed as a ratio of WT levels. Values are expressed as mean ±SEM. *, P<0.05.

Complex II has a unique dual role in mitochondrial metabolism: it is both a complex in ETC and an enzyme in the TCA cycle. SDHA catalyzes the oxidation of succinate to fumarate and electrons generated by this reaction are transferred through iron-sulfur clusters in SDHB and on to the inner-membrane subunits, SDHC and SDHD, where they are delivered to the quinone pool of the ETC. Consequently, the complex has two distinct enzymatic activities: succinate oxidation (SDH activity) and electron transfer (complex II activity). A previous study detected diminished complex II activity in SIRT3 KO liver mitochondria [17], but the effect of SIRT3 on SDH activity has not been shown. Because SDHA is the only known acetylated subunit of complex II, we focused specifically on SDH activity. We found that SIRT3 KO MEFs had a 25% reduction in SDH activity (Fig. 4A). Next, we isolated mitochondria from SIRT3 WT and KO mice to determine whether SIRT3 could influence SDH activity *in vivo*. We observed no difference in SDH activity in liver mitochondria (Fig. 4B), consistent with a previous report [15]. However, when we measured SDH activity in brown adipose tissue (BAT), a highly oxidative tissue in which several groups have reported functional roles for SIRT3 [9], [24]–[26], we found a 30% reduction in SDH activity in SIRT3 KO mice (Fig. 4C). These results suggest that acetylation of SDHA can affect SDH activity *in vivo*, perhaps in a tissue- or context-specific manner.

## Discussion

In this study, we identified subunits of complex II and complex V as SIRT3 interacting proteins. The SDHA subunit of complex II was previously shown to be hyperacetylated in SIRT3 KO mice. We further examined SDHA, demonstrating that SIRT3 can directly deacetylate SDHA and that succinate dehydrogenase activity is reduced in SIRT3 KO cells and BAT from SIRT3 KO mice. We found that the entire peripheral surface of SDHA can be acetylated, implying that regulation of mitochondrial proteins by acetylation may not always be as simple as modification at a single residue triggering a change in activity. Moreover, despite our specific analyses of SDHA acetylation, we did not detect acetylation at three previously-identified residues: K423, K517 and K538 [18], [19]. It is possible that unidentified acetylated lysines still remain to be discovered. Alternatively, acetylation could be sequence-independent, designed to modify the surface of SDHA rather than a particular residue. It will be interesting for future studies to determine how acetylation influences SDH activity, the regulation of SDH acetylation, and the specific residues or domains that are involved.

Participating in both the TCA cycle and electron transport chain, complex II is uniquely well situated to coordinate flux through both pathways. Deacetylation of SDHA by SIRT3 could therefore potently stimulate mitochondrial oxidative capacity in response to nutrient stress. For example, CR induces SIRT3 expression [26] and dramatically reduces mitochondrial acetylation in BAT [18], a tissue in which SIRT3 regulates SDH activity. CR-mediated induction of SIRT3 in BAT could facilitate SDHA deacetylation and activation. Somewhat counter intuitively, however, we and others found that CR increases SDHA acetylation in the liver [18]. As we did not see an effect of SIRT3 expression on liver SDH activity, it is possible that acetylation of SDHA confers alternative effects in liver, perhaps by regulating formation of mitochondrial supercomplexes to facilitate electron transfer or other complex II-protein interactions to influence substrate channeling. It will be interesting for future studies to address how diet and tissue-specific changes in acetylation influence SDHA function.

Intriguingly, some acetylation sites are in close proximity to residues that are mutated in human disease. For example, the R554W mutation in humans reduces SDH activity and is associated with Leigh's syndrome [27]. R554 is near the substrate entrance channel and mutation in this residue could affect substrate entry or exit [21]. As K550 is on the same unstructured loop, it is possible that K550 could also influence SDH activity in a similar manner. Future studies may reveal new roles for mitochondrial acetylation in human mitochondrial disorders.

The prevalence of acetylation on mitochondrial proteins—particularly enzymes involved in metabolism—attests to the importance of this post-translational modification. Indeed, dysregulation of the mitochondrial deacetylase SIRT3 is already known to drive pathological states such as dilated cardiomyopathy and tumorigenesis in mice [25], [28]–[30]. Elucidation of the specific consequences of mitochondrial protein acetylation, as well as identification of the relevant acetyltransferases and deacetylases, will be critical to our understanding of mitochondrial biology. It is possible that in the future, directed modulation of mitochondrial protein acetylation will hold therapeutic promise for these diseases.

## Materials and Methods

### Cell culture, plasmids and transfection

SIRT3 WT and KO mouse embryonic fibroblasts (MEFs) were previously described [25]. HEK293T (ATCC) cells were cultured in DMEM supplemented with 10% FBS, penicillin/streptomycin and 2 mM L-glutamine; MEFs were cultured with an additional 0.1 mM beta-mercaptoethanol and non-essential amino acids. Cells were transfected with expression plasmids using Fugene 6 transfection reagent (Roche). SIRT1-7 plasmids with C-terminal FLAG-tags were previously described [31], [32].

### Animal studies

Animal studies were performed according to protocols approved by the Institutional Animal Care and Use Committee, the Standing Committee on Animals at Harvard. Male 129Sv SIRT3 WT and KO [4] littermates (a generous gift from Dr. Fred Alt) were fed a normal chow diet (PicoLab diet 5053). For calorie restriction (CR) experiments, 12-week old male C57BL/6 mice (Jackson Laboratories) were placed on a stepwise 3 month 40% CR regimen according to a modified version of an established protocol [33].

### Immunoprecipitation and immunoblotting

FLAG immunoprecipitations (IPs) were performed as described [25]. For all other IPs, mitochondria were isolated from liver of SIRT3 WT or KO mice according to established methods [34]. Acetylated proteins were purified using anti-acetyl-lysine antibody bound to protein G agarose (Invitrogen), with 10 mM nicotinamide and 400 nM trichostatin A added to the IP buffer. A complex II immunocapture kit (MitoSciences) was used to purify complex II from isolated mitochondria according to manufacturer instructions. Immunoprecipitations with anti-GFP antibodies were used for negative controls. The following antibodies were used: Total OXPHOS antibody cocktail, complex II antibody cocktail and OSCP (Mitosciences); Flag (Sigma); SIRT3 and acetyl-lysine (Cell Signaling); GFP (Santa Cruz), and GDH (USBiological).

### Mass spectrometry

To determine proteins interacting with SIRT3, FLAG IPs were performed as described above on cells expressing empty vector or SIRT3-FLAG. After washing, IPs were eluted with FLAG peptides, disulfide bonds were reduced with 5 mM DTT and cysteine residues alkylated with 15 mM iodoacteamide. Proteins were precipitated by adding trifluoro acetic acid (TCA, 25%, w/w), then resuspended in 1 M urea and 50 mM Tris (pH 8.5) and digested by incubation with 1.6 µg trypsin (seuquencing grade, Promega) at 37°C for 12 hours. Peptides were desalted using C_18_ StageTips [35]. To map the acetylation sites on SDHA, complex II was immunoprecipitated as described above, run on an SDS-PAGE gel and stained with Coomassie blue. The 70 kDa band corresponding to SDHA was excised and treated with DTT and iodoacetamide. The protein was then digested in-gel using trypsin, and peptides were desalted as described above. Both samples were analyzed using nano-scale microcapillary C18 liquid chromatography (LC-MS/MS) on either an LTQ Orbitrap XL or an LTQ Orbitrap Velos mass spectrometer (Thermo Scientific) equipped with an Agilent 1100 binary HPLC pump (Agilent, Santa Clara, CA) essentially as described previously [36]. MS/MS spectra were assigned using the SEQUEST algorithm by searching them against a target-decoy protein sequence database based on the mouse IPI database (v 3.60) to which protein sequences of SIRT3-FLAG and of common contaminants were added. Enzyme specificity was set to tryptic, cysteine residues were searched as carbamidomethylated, methionine residues were allowed to be oxidized, lysine residues were allowed to be acetylated for the acetylation site mapping and up to 2 or 4 (acetylation site mapping) missed cleavages on tryptic cleavage sites were accepted. All peptide assignments were filtered to a false-discovery rate (FDR) of smaller than 1% by applying the target-decoy database search strategy [37] using a linear discrimination analysis method as described previously [38]. The resulting MS/MS data set was filtered further to obtain an protein identification FDR of smaller than 1% [38]. MS/MS spectra of acetylated SDHA peptides were manually validated.

### Deacetylation reactions

Recombinant SIRT3 was purchased from Biomol. His-tagged SIRT3 and SIRT3-H248Y were purified from *E. coli* as described [5]. Complex II was immunoprecipitated from SIRT3 KO liver mitochondria and incubated for 2 hours at 37°C in SDAC buffer (50 mM Tris·HCl (pH 9.0), 4 mM MgCl_2_, 50 mM NaCl, 0.5 mM DTT) in the presence or absence of recombinant enzyme, 1 mM NAD or 10 mM nicotinamide.

### SDH activity assays

SDH activity was measured from purified liver or brown adipose tissue mitochondria resuspended to a final concentration of 1 mg/ml in buffer B (280 mM sucrose, 10 mM Tris, pH 7.4). 30 µg mitochondria were used to assay dichlorophenolindophenol (DCPIP) reduction in 1.0 mL of 50 mM 50 mM KH_2_PO_4_ (pH 7.5), 1.5 mM KCN, 1 mM PMS and 17.5 mM succinate. Malonate-sensitive SDH activity was measured in MEFs that were quickly freeze-thawed in liquid nitrogen and resuspended in buffer A (250 mM sucrose, 20 mM Tris, 2 mM EDTA, 1 mg/ml BSA, pH 7.2). DCPIP reduction was measured on 5×10^5^ cells as for mitochondria, with the addition of 0.2 mM thenoyltrifluoroacetone to the reaction mixture. Samples were measured in duplicate against a reference cuvette containing 10 mM sodium malonate. The rate of DCPIP reduction was measured by the decrease in absorption at 600 nm.

### Structural modelling

Residues corresponding to the mouse acetylated lysines were mapped onto avian complex II (PDB 2H89) [39]. Molecular graphics images were produced using the UCSF Chimera package from the Resource for Biocomputing, Visualization, and Informatics at the University of California, San Francisco (supported by NIH P41 RR001081) [40].

## Supporting Information

Figure S1 **CR increases SDHA acetylation.** The relative abundance of acetylation at specific lysine residues was assessed by semiquantitative mass spectrometry.
(TIF)

Table S1 SDHA immunopurified from liver mitochondria of control (C), calorie restricted (CR), SIRT3 wild-type (WT) or knock-out (KO) mice that were fed or fasted for 48 h was analyzed for acetylation by mass spectrometry. The 13 identified sites are indicated by ‘x’. The 6 novel sites are shown in red. Sites previously identified by proteomic studies of mouse liver mitochondria are shown in white, with appropriate citation. Sequences of SDHA were aligned using the Clustal W algorithm and are shown in green (conserved lysine) or yellow (not conserved).
(XLS)

Appendix S1 **SDHA is hyperacetylated in SIRT3 KO mice.** (A) Additional exposures of Western blot of complex II IP described in Fig. 3A. (B) Western blot of the input from the complex II IP illustrating hyperacetylation of several mitochondrial proteins in SIRT3 KO liver. AcK, acetyl-lysine.
(TIF)
