# Supplementary figures and images for: Succinate Dehydrogenase Is a Direct Target of Sirtuin 3 Deacetylase Activity
Source: PLoS One. 2011 Aug 17;6(8):e23295. doi: 10.1371/journal.pone.0023295 (PMC3157345; doi:10.1371/journal.pone.0023295)

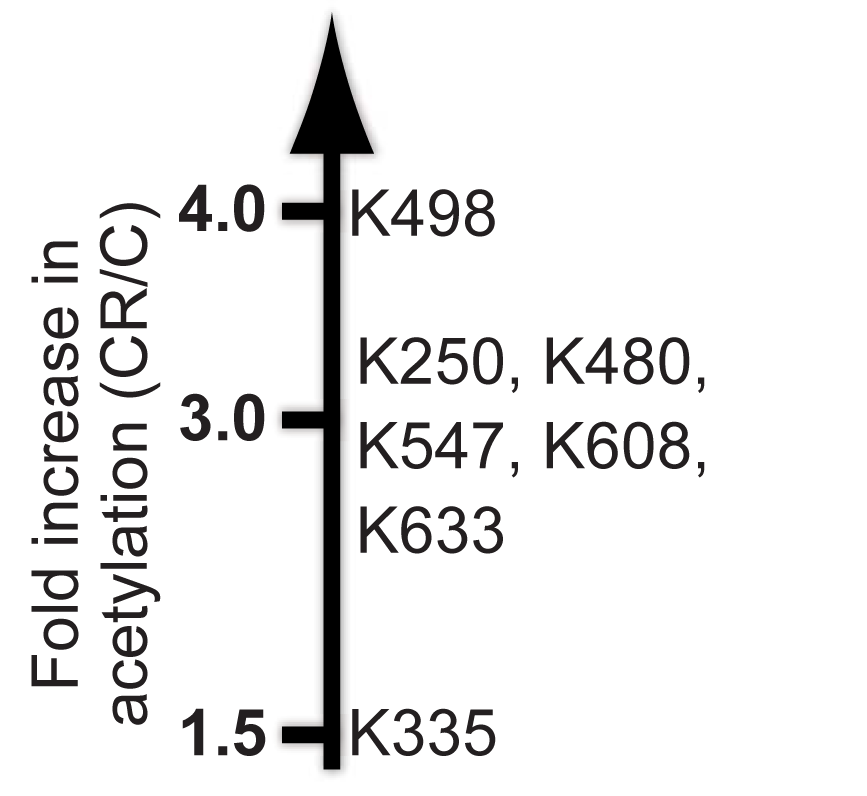

Supplement: Figure S1 — CR increases SDHA acetylation. The relative abundance of acetylation at specific lysine residues was assessed by semiquantitative mass spectrometry. (TIF) [file pone.0023295.s001.tif]

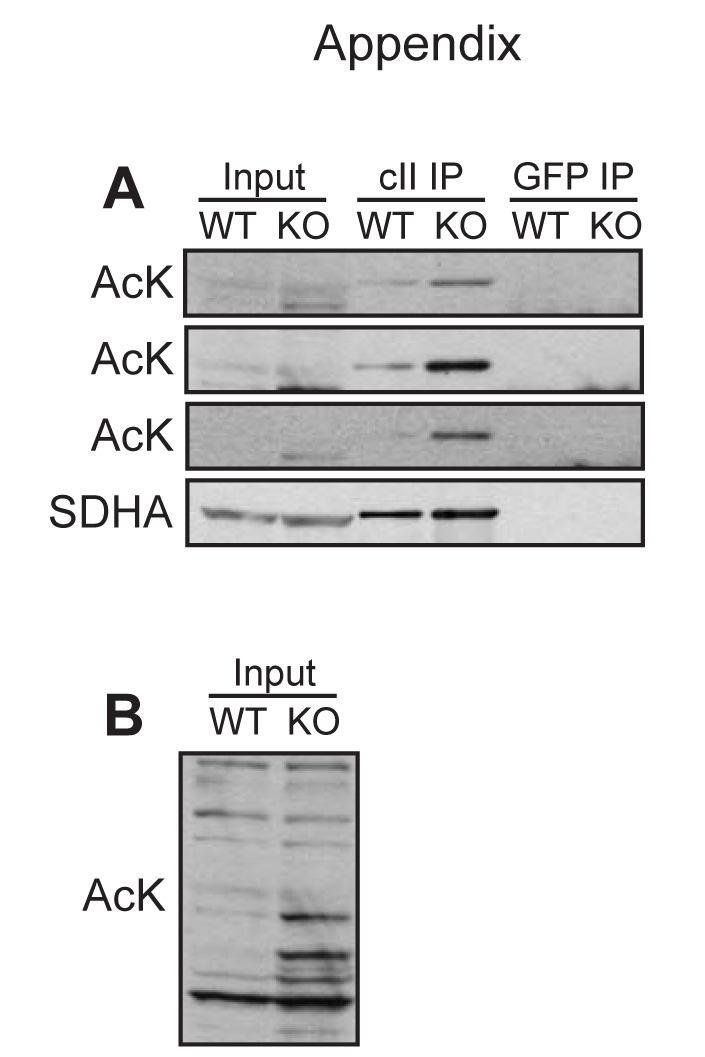

Supplement: Appendix S1 — SDHA is hyperacetylated in SIRT3 KO mice. (A) Additional exposures of Western blot of complex II IP described in Fig. 3A. (B) Western blot of the input from the complex II IP illustrating hyperacetylation of several mitochondrial proteins in SIRT3 KO liver. AcK, acetyl-lysine. (TIF) [file pone.0023295.s003.tif]
